# Highly Sensitive, Stretchable Pressure Sensor Using Blue Laser Annealed CNTs

**DOI:** 10.3390/nano12132127

**Published:** 2022-06-21

**Authors:** Chanju Park, Munsu Choi, Suhui Lee, Hyunho Kim, Taeheon Lee, Mohammad Masum Billah, Byunglib Jung, Jin Jang

**Affiliations:** Advanced Display Research Center (ADRC), Department of Information Display, Kyung Hee University, 26 Kyungheedae-ro, Dongdaemun-gu, Seoul 02447, Korea; cjpark@tft.khu.ac.kr (C.P.); mschoi@tft.khu.ac.kr (M.C.); shlee5@tft.khu.ac.kr (S.L.); hhkim@tft.khu.ac.kr (H.K.); thlee@tft.khu.ac.kr (T.L.); masum@tft.khu.ac.kr (M.M.B.); bljung@tft.khu.ac.kr (B.J.)

**Keywords:** pressure sensor, carbon nanotube (CNT), blue laser annealing (BLA)

## Abstract

A piezoresistive sensor is an essential component of wearable electronics that can detect resistance changes when pressure is applied. In general, microstructures of sensing layers have been adopted as an effective approach to enhance piezoresistive performance. However, the mold-casted microstructures typically have quite a thick layer with dozens of microscales. In this paper, a carbon microstructure is formed by blue laser annealing (BLA) on a carbon nanotube (CNT) layer, which changes the surface morphology of CNTs into carbonaceous protrusions and increases its thickness more than four times compared to the as-deposited layer. Then, the pressure sensor is fabricated using a spin-coating of styrene–ethylene–butylene–styrene (SEBS) elastomer on the BLA CNTs layer. A 1.32 µm-thick pressure sensor exhibits a high sensitivity of 6.87 × 10^5^ kPa^−1^, a wide sensing range of 278 Pa~40 kPa and a fast response/recovery time of 20 ms, respectively. The stability of the pressure sensor is demonstrated by the repeated loading and unloading of 20 kPa for 4000 cycles. The stretchable pressure sensor was also demonstrated using lateral CNT electrodes on SEBS surface, exhibiting stable pressure performance, with up to 20% stretching.

## 1. Introduction

The pressure sensor is of increasing interest because of its various applications, such as object detection, display touch, electronic skin (e-skin), etc. [1,2,3]. Mobile and healthcare devices with touch sensors are used in human activities [4,5]. The demand for the development of pressure sensors, converting the external stimulus into electrical signals, is continuously increasing [6,7,8]. In general, the pressure sensors can be classified as piezoresistive [9,10,11,12,13,14,15,16], capacitive [17,18,19,20,21,22,23], and piezoelectric types [24,25,26,27,28].

Among them, piezoresistive-type pressure sensors operate on a simple principle that detects the resistance change under applied pressure [14,15,16]. Usually, conductive fillers such as carbon nanotubes (CNTs) [9,10,14], graphenes [29], nanoparticles [30,31,32], and nanowires [16,33] have been used to fabricate piezoresistive pressure sensors. However, polymer-composite-film-based piezoresistive pressure sensors typically have the disadvantage of low sensitivity. Therefore, pressure sensors with porous structure [17,34], microstructure [13,35,36,37], and pyramid structure [38,39,40,41] have been developed to achieve improved sensitivity and response time. The microstructure efficiently modulates the contact resistance and improves the response and recovery speed and the stability of the pressure sensor. A piezoresistive pressure sensor with high sensitivity over 1000 kPa^−1^ typically has high initial resistance and large pressure-sensitive current deviation, which are essential requirements for high sensitivity [36,37,40,41]. Recently, ultra-high-sensitive pressure sensors with sensitivities of 10^5^–10^6^ kPa^−1^ were demonstrated [42,43,44]. The strategy for achieving ultra-high sensitivity is based on a microstructure sensing layer. Li et al. achieved maximum sensitivity as high as 3.8 × 10^5^ kPa^−1^ using double-layered Au-coated polydimethylsiloxane (PDMS) sensing layers [43]. The high sensitivity was attributed to a double layer of Au-coated interlocked PDMS film and an interlocked structure. Lee et al. showed a pressure sensor with a high sensitivity of 3.8 × 10^5^ kPa^−1^ using a gradient-resistance pressure sensor with a single sensing layer [42]. With the combination of three different gradient-resistance layers using Poly(3,4-ethylenedioxythiophene):poly(styrene sulfonate) (PEDOT:PSS) on a microdome sensing layer and a silver nanowire (AgNW)/Pt-coated microdome electrode, the ultra-high-sensitive pressure sensor could be achieved. Jung et al. showed a single micro-structured sensing layer with contact resistance engineering using a semi-insulating contact region and a highly conductive path region [44]. By selectively coating semi-insulating materials on a microstructure contact region, piezoresistive characteristics could be modulated. To achieve ultra-high sensitivity of over 10^5^ kPa^−1^, most sensors have adopted a microstructured mold with conductive materials. However, the microstructures are thick with dozens of micrometer scales, and could be made using a complicated process including PDMS casting and a conductive layer coating and lamination process [38,45,46].

The laser irradiation techniques that make porous structures of multi-walled (MW) CNTs/Polyamic acid (PAA) [45], polyimide (PI) [46], and PDMS [47] were applied to enhance the sensitivity. For example, the sensitivity of the pressure sensor is not high, because the laser-induced graphene (LIG) has low resistivity, and the resistance change with pressure is small. Approaches to making porous layer for sensors, including porous PDMS sponge [48], PI/CNTs aerogel [49], and foaming agent [50] were proposed. However, the pore size in the sensing layer depends on the materials, such as microspheres and foaming materials, which need additional processes, and the resulting film is quite thick, from 10 μm to 1 cm. Compared to previous studies, the BLA technique could be cost effective and used for a large area with a very thin sensing layer.

A previous study reports on a single-walled CNT (SWNT) pressure sensor on a very thin alumina membrane [51]. By etching the 300 μm-thick Si wafer, the remaining 10 μm Si and 100 nm Al_2_O_3_ were used as a backplane. Then, a single SWNT was placed on the membrane to detect pressure from the backplane. The previous work reports the SWNT on a very restricted area membrane using a 300 μm-thick Si etch process. The most important point of this work is the large area capability. Our method is very simple and has no limit in the substrate area. Spray-coating and the BLA process can cover a large area of over 100 cm × 100 cm. In addition, the BLA CNTs/styrene–ethylene–butylene–styrene (SEBS) pressure sensor has the current change from 10 pA to 20 μA within 40 kPa, and the maximum sensitivity can be 6.9 × 10^5^ kPa^−1^.

In this study, the pressure sensing layer was prepared via BLA of a CNTs layer [52]. The laser irradiation on CNTs’ thin film not only changes the surface morphology of CNTs into carbon mass protrusions, due to laser-induced sudden elevation of local temperature [53], but reduces the defective CNTs, which is demonstrated by I_D_/I_G_ ratio reduction with increasing blue laser energy density by Raman spectra [54]. The morphology of BLA CNTs changes remarkably at 5 J cm^−2^. Several reports indicate the effect of laser annealing on CNTs to reduce defective CNTs and amorphous carbons by high surface temperature [55,56]. We have demonstrated that laser annealing can change surface morphology by a rapid increase in temperature caused by BLA, which can be seen from the SEM images and Raman analysis. The morphology at the top region is uniformly changed by BLA. Then, this BLA CNT layer is coated with SEBS elastomer to fabricate an ultra-thin pressure sensor. The laser-induced microstructure of CNTs thin film can effectively reduce the fabrication process compared to a conventional mold casting-based microstructure. The fabricated pressure sensor exhibits a wide sensing range of ~40 kPa, with a high sensitivity of 1.32 × 10^6^ kPa^−1^. It shows the fast rising and falling time of 20 ms, with excellent mechanical stability against repeated loading and unloading tests under high pressure of 20 kPa for 3600 cycles. Finally, a stretchable BLA CNT/SEBS sensor is demonstrated with a pressure sensor on a rigid polyimide (PI) substrate and a stretchable CNT electrode. The piezoresistive characteristics do not degrade at up to 20% of strain with the stretchable CNT electrode. In addition, the pressure sensor successfully distinguishes the bending degree on attachment to the finger joint.

## 2. Materials and Methods

### 2.1. Materials

Multi-walled carbon nanotubes of 3 wt% with diameters of 5~15 nm in water dispersions were purchased from US Research Nanomaterials, Inc. (Houston, TX, USA). Graphene oxide, SEBS Tuftec^TM^ H1052, and toluene were purchased from Grapheneall (Hwaseong-si, Korea), Asahi Kasei Corp (Tokyo, Japan), and Sigma-Aldrich (St. Louis, MO, USA). All of the chemicals were used without further purification.

### 2.2. Fabrication of BLA CNT/SEBS on PI

The process flow of CNTs coating and BL exposure on the CNTs layer for the pressure sensor can be seen in Figure S1. CNTs/graphene oxide (GO) for the release layer was spray-coated onto the carrier glass using a mixture of CNT/GO solution to detach the PI substrate from the carrier glass [57,58]. The very thin CNT/GO layer was soft baked at 130 °C for 15 min in air and then hard-baked at 290 °C for 2 h in a vacuum oven. The PI was spin-coated on glass and baked at 450 °C for 2 h in N_2_ atmosphere. A buffer layer of SiN_x_/SiO_2_ was deposited on the PI substrate at the substrate temperature of 420 °C. The SiO_2_ layer was treated with UV/O_3_ for 300 s for spray-coating with CNTs and annealed at 290 °C for 2 h in a vacuum. The CNTs layer was exposed by blue laser (beam size: 520 µm × 20 µm, laser energy density 5.06 J cm^−2^). After BLA on CNTs, the 60 mg mL^−1^ SEBS solution diluted in toluene was spin-coated and cured at 120 °C for 10 min.

## 3. Results and Discussion

### 3.1. Thin-Film Analysis of Blue Laser Annealing on CNTs

**Figure 1:**
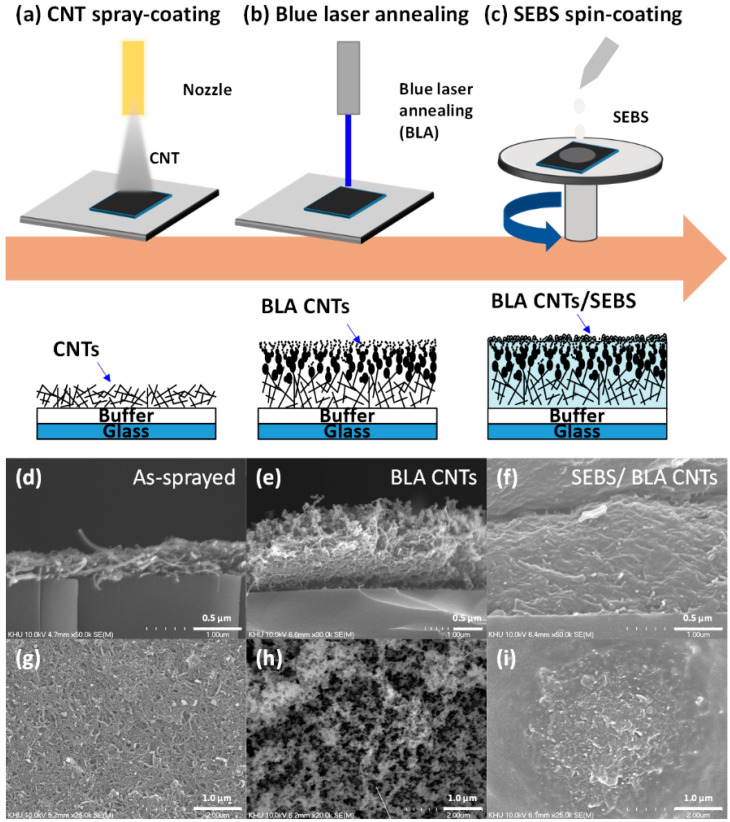
Schematic and cross-sectional illustration of fabrication process of the BLA CNTs/SEBS pressure sensor: (**a**) spray-coating of CNTs; (**b**) blue laser annealing; (**c**) SEBS coating on BLA CNTs layer. Cross-sectional and top view of SEM images of: (**d**); (**g**) as-sprayed CNTs; (**e**,**h**) BLA CNTs; (**f**,**i**) SEBS coated BLA CNTs.

Figure 1a–c show a schematic illustration of the fabrication process of BLA CNT/SEBS film. The CNTs were deposited by spray-coating a uniform CNTs layer of 330 nm. Then, BL irradiation on the CNTs layer was performed to change the CNTs’ surface morphology into carbonaceous protrusion with high thickness deviation. Note that high laser energy can elevate local temperature of the CNTs surface, which can transform the CNTs morphology into the large aggregation of carbons [53]. The BL has a fluence of 5.05 J cm^−2^ with a 520 × 20 µm^2^ laser beam size. Finally, the SEBS in toluene of 60 mg mL^−1^ was spin-coated onto BLA CNTs to control the piezoresistive characteristics and to provide the elasticity of the pressure sensor with high durability against mechanical stress. Note that controlling the thickness of the thin SEBS insulating layer is important for lowering the initial current (I_0_) to achieve an ultrahigh sensitivity. The cross-sectional and top view of the scanning electron microscopic (SEM) images with each process step are shown in Figure 1d–i. The thickness of the as-sprayed CNTs layer is 330 nm, which is uniform over the substrate and increases, by almost four times, to over 1.32 μm upon BLA due to carbon protrusions caused by BL absorption. It can be clearly observed that the surface morphology of the CNTs layer changes significantly after BL exposure. The morphology change of the CNTs layer can be seen as a function of the incident BL energy density in Figure S2. By increasing the laser energy density up to 5.06 J cm^−2^, the top region of the CNTs layer changes its morphology to carbon protrusions, due to the rapid heating of CNTs. The carbon–carbon bonds in CNTs are broken by BL irradiation, and the broken C atoms aggregate with the C atoms [53]. BLA-induced change in carbon microstructures can eliminate the conventional mold casting method of microstructure fabrication, which could be one of the difficulties for large-area sensor devices.

**Figure 2:**
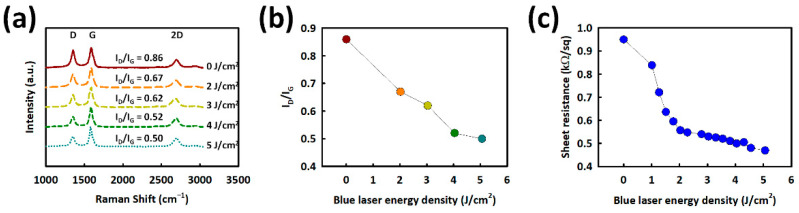
(**a**) Raman spectra; (**b**) ID/IG ratio of BLA CNTs layer with blue laser energy densities. (**c**) The sheet resistance of the BLA CNTs as a function of BL energy density.

The effect of BLA on CNTs with different laser-energy densities, from 0 to 5 J cm^−2^, was analyzed with Raman spectra, as shown in Figure 2a. Two dominant peaks of BLA CNTs at 1350 and 1590 cm^−1^ can be seen, corresponding with the D- and G-bands of graphitic carbon. The intensity ratio of D- to G-band (I_D_/I_G_), which can evaluate the defects in BLA CNTs, decreases from 0.86, to 0.67, 0.62, 0.52, and 0.50 with increasing BL energy from 0, to 2, 3, 4, and 5 J cm^−2^, as shown in Figure 2b. These results confirm that the BLA can effectively reduce the defect states in the CNTs layer [54]. In addition, the sheet resistance of BLA CNTs decreases with increasing BL exposure energy, as shown in Figure 1 h, from 955 ohm sq^−1^ to less than 490 ohm sq^−1^, when the BL energy density increases to 5.06 J cm^−2^. The reduction in sheet resistance could be related to a defect reduction in CNTs caused by BLA, as shown in Figure 2b. The I_D_/I_G_ ratio decreases significantly from 0.86 (without BLA) to 0.52 (4 J cm^−2^), and it tends to saturate to 0.50 (at 5 J cm^−2^), from a Raman analysis, as shown in Figure 2. The sheet resistance shows a similar trend. The I_D_/I_G_ ratio appears to saturate at a higher laser intensity.

**Figure 3:**
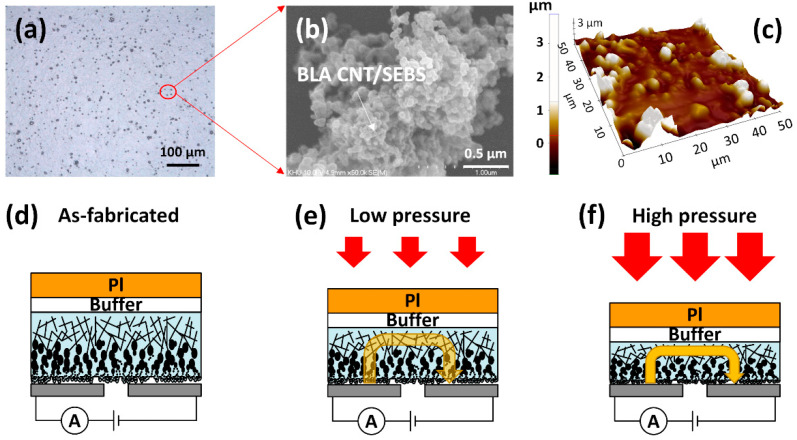
(**a**) Optical, (**b**) SEM, and (**c**) AFM images of the BLA CNTs/SEBS film. Schematic illustration of the working mechanism of pressure sensor. Mechanism of the pressure sensor: the schematic views of the operation of BLA CNTs/SEBS pressure sensor under (**d**) without external pressure (278 Pa~3.8 kPa), (**e**) low external pressure, and (**f**) high external pressure (9.3 kPa~38.9 kPa). The arrow indicates the current flow.

Figure 3a shows the optical image of the BLA CNTs/SEBS film. The BLA CNTs coated with SEBS show the microstructure islands made of carbonaceous protrusions. Figure 3b shows the SEM image of protrusive BLA CNT/SEBS over a SEBS floor. The height of protrusions is ~3 μm from the AFM image, as shown in Figure 3c. The BLA CNTs/SEBS film on polyimide (PI) substrate was made to characterize the piezoresistive behavior. The PI substrate was detached from carrier glass, followed by being flipped over and laminated onto a lateral electrode, as shown in Figure 3d. The PI substrate is used to protect BLA CNTs/SEBS film against damage by tear or scratch from external force. Figure 3e,f show the schematic illustration of the working mechanism of a pressure sensor with low and high pressures, respectively. Without external force, the current is very low, at less than 10^−12^ A, because very few BLA CNTs/SEBS protrusions have contact with electrodes. In the low-pressure region, the current starts to flow between electrodes through BLA CNTs/SEBS by an increased contact region, as shown in Figure 3e. High sensitivity at low pressures can be achieved by causing a large resistance change through the contact of BLA CNTs/SEBS protrusions to the electrode. However, the current could be saturated in a high-pressure region over 9.3 kPa, as shown in Figure 3f. Generally, the sensitivity of the pressure sensor depends on the pressure range. The sensitivity of the proposed pressure sensor depends on the contact region between the sensor material and the electrodes.

### 3.2. Piezoresistive Characteristics of BLA CNTs/SEBS Pressure Sensor

**Figure 4:**
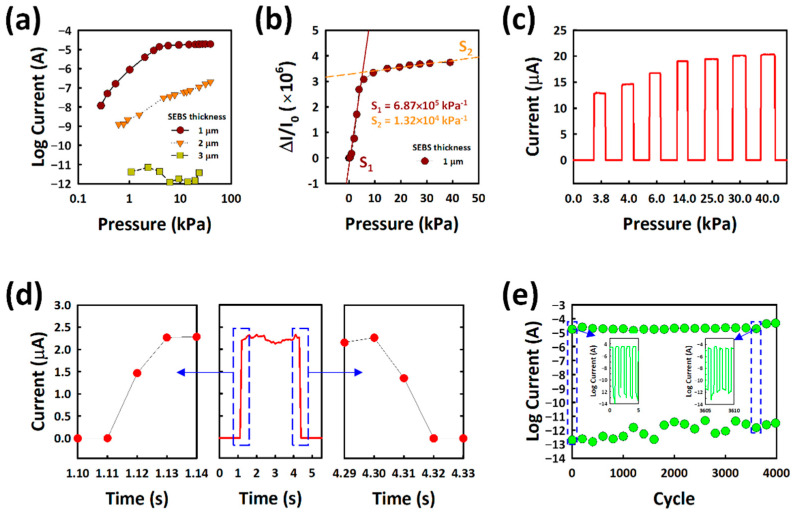
Electrical characteristics of BLA CNT/SEBS pressure sensor. (**a**) piezoresistive characteristics of a pressure sensor with SEBS thickness variation. (**b**) Relative current change on 1 μm-thick SEBS pressure sensor as a function of pressure at V = 0.1 V. Sensitivities (S_1_, S_2_) were extracted in low- and high-pressure regions. (**c**) Real-time measurements of current changes under different pressures. (**d**) The response and recovery times of the pressure sensor. (**e**) Durability test of the pressure sensor until 4000 cycles at 20 kPa.

We changed the SEBS thickness on the BLA CNTs layer to see the effect of an insulating SEBS layer on the pressure sensor. To see the effect of SEBS thickness on BLA CNTs on the performance of the pressure sensor, different concentrations of SEBS in toluene were spin coated on BLA CNTs to control the insulation thickness. Figure 4a shows the piezoresistive characteristics depending on the SEBS thickness with external pressure. For a 1 μm-thick SEBS case, the current is 11.7 nA at 278 Pa and saturates to 20 μA at 40 kPa. The current at zero pressure is 5.2 pA. Thus, the current increases from 5.2 pA to 11.7 nA by 2000 times at 278 Pa, and from 5.2 pA to 20 μA by 3.8 × 10^6^ times at 40 kPa. However, with increasing SEBS thickness from 1 μm to 3 μm, the piezoresistive performance decreases dramatically. At a 2 μm-thick SEBS case, the sensor exhibits 1.23 nA at 620 Pa, which is a relatively lower current at the same pressure response compared with the 1 μm-thick SEBS pressure sensor. It can be seen that the thicker SEBS is surrounded by BLA CNTs, and fewer protrusions are shown in Figure S3b. When the SEBS thickness is over 3 μm, there is no current response even at high pressure, due to the thick SEBS insulating layer, as shown in Figure S3c.

To calculate the sensitivity of BLA CNTs with 1 μm-thick SEBS pressure sensor, Figure 4b shows the relative current changes (ΔI/I_0_) plotted as a function of applied pressure. The sensitivity (S) is calculated by the following equation:

S = Δ(δI/I_0_)/(δP), (1)

The BLA CNT/SEBS sensor shows a broad sensing range (278 Pa~40 kPa) with a very high sensitivity of 6.87 × 10^5^ kPa^−1^ in the low-pressure region 278 Pa~3.89 kPa, and 1.32 × 10^4^ kPa^−1^ in the high-pressure region 5.7 kPa~38.9 kPa, respectively. High sensitivity can be achieved due to the extremely low initial current and high current under applied external pressure.

We compare the sensor performances and structures reported in the literature shown in Table 1 to our proposed device. Note that the BLA CNTs/SEBS sensor has high sensitivity even within a single-layer structure without any microstructure formation. Therefore, it can be easily applied to mobile electronic systems.

Figure 4c shows the real-time sensor performance at the pressure of 2.5~40 kPa. The measurements were performed with and without loading pressure on the sensor. The result indicates that the sensor exhibits an excellent sensing performance. The current starts to saturate above 14.8 kPa. Figure 4d shows the response and recovery times of the pressure sensor, with faster response and recovery times of 20 ms. The mechanical durability of the sensors was tested by loading and unloading a pressure of 20 kPa at V = 0.1 V for 4000 cycles. Figure 4e shows the stable current even changes at a high pressure of 20 kPa, until 4000 cycles.

**Figure 5:**
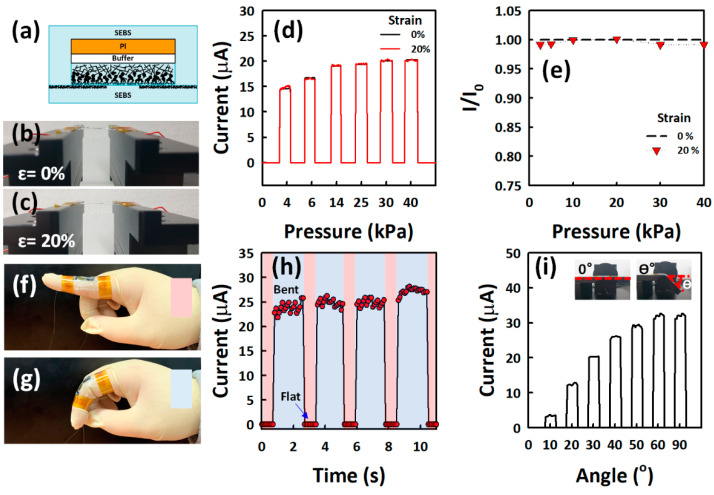
Performances of the stretchable BLA CNTs pressure sensor. (**a**) Cross-sectional view of the stretchable pressure sensor. Photographs of a stretchable pressure sensor at (**b**) 0% and (**c**) 20% strain. (**d**) Current change of the stretchable pressure sensor by loading a rubber block under 0% and 20% stretching, respectively. (**e**) Normalized currents as a function of external pressure, measured at 0% and 20% strain, respectively. I0 is the initial current before stretching, and I is the current measured after applying an external strain of 20% stretching. Photographs of stretchable pressure sensor attached on the finger at (**f**) flat and (**g**) bent state. (**h**) The current change of the stretchable pressure sensor on finger under flat state (red) and bent state (blue). (**i**) The current change of the stretchable pressure sensor under bending of the angle between 0 and 90°.

To apply BLA CNT/SEBS films on stretchable electronics, BLA CNT/SEBS on PI film was laminated on CNT electrodes, as shown in Figure 5a. The detailed process flow for stretchable BLA CNT/SEBS pressure sensors is shown in Figure S4. Figure 5b,c show the photographs of stretchable pressure sensor at the initial and 20% stretched states, respectively, using a stretchability-measurement machine. Figure 5d shows the current change of the pressure sensor with and without strain. Note that the stretchability of pressure sensor could be achieved with a combination of the pressure sensor on a flexible PI island transferred onto a SEBS substrate and stretchable CNT electrodes. Figure 5e shows the relative current changes of the pressure sensor plotted as a function of strain. It demonstrates that the stretchable pressure sensor maintains its piezoresistive characteristics at 20% strain. The stretchable pressure sensor was mounted to the joint of a finger to mimic the motions of the human body, as shown in Figure 5f,g, for the real-time current measurement. At a flat state (Figure 5f), the sensor exhibits a stable low current state of 30 nA. At finger bent (Figure 5g), the current sharply increases to 25 μA, which corresponds to the high-pressure region (>20 kPa). Figure 5i shows the current changes in the stretchable pressure sensor under different bending angles. The results show that the current increases and reaches the maximum of 30 μA at 60°. This is due to the pressure applied on the CNT/SEBS film by bending.

## 4. Conclusions

In summary, a highly sensitive pressure sensor is demonstrated using BLA CNTs by laser irradiation on the CNTs layer. The BLA CNTs were coated with SEBS elastomer, resulting in a very thin, highly sensitive pressure sensor. The sensor is capable of operating in the wide pressure range from 278 Pa to 40 kPa. The pressure sensor shows a high sensitivity of 6.87 × 10^5^ kPa^−1^ at a pressure range of 278 Pa~3.89 kPa and a high current ratio of >2000 at a pressure of 278 Pa. The pressure sensor has a fast rising/falling time of 20 ms and excellent durability, with repeated loading and unloading tests under 20 kPa for 4000 cycles. When it is made with CNTs lateral electrodes on SEBS substrate, it maintains its piezoresistive characteristic until 20% stretching. Therefore, a BLA CNT/SEBS pressure sensor can be applied to ultra-thin, large-area e-skin sensors.

## Figures and Tables

**Table 1:** Summary of the performances of the pressure sensors with a high sensitivity of over 1000 kPa^−1^ reported in the literature.

Structure	Pressure Range (Pa)	Sensitivity (kPa^−1^)	Response/Recovery Time (ms)	Sensor Thickness (μm)	Fabrication Method	Reference
Polypyrrole/PDMS micropyramid	0.075–1 k	1.9 × 10^3^ 4.6 × 10^2^	0.05/6.2	>20	Mold casting	[40]
Sandpaper-molded rGO/PDMS	10–400 k	1.1 ×10^3^	150/40	>500	Mold casting	[36]
cellulose/nanowire nanohybrid network	100–150 k	>5 × 10^3^	-/<1	~1	Spray-coated cellulose	[37]
Au/PDMS micropyramid and PEDOT:PSS/Au/PI	0.425–2 k	9.2 × 10^2^ 1.2 × 10^4^ 1.5 × 10^3^	0.44/0.08	-	Mold casting	[41]
Au/PMDS pyramids	0.25–56 k	3.8 × 10^5^ 2.7 × 10^5^ 4.9 × 10^4^	75/50	555	Mold casting	[43]
PEDOT:PSS/PUD interlocked with TPU electrode	0.025–100 k	~3.8 × 10^5^	0.016/-	~5	Mold casting	[42]
Ag nanocrystal/PDMS janus-like pyramid	220–3 k	1.9 × 10^4^ 1.6 × 10^6^	-	>250	Mold casting	[44]
BLA CNT/SEBS	278–40 k	6.9 × 10^5^ 1.3 × 10^4^	<20/<20	~1	BLA CNT	This work

## Data Availability

The data are available upon request to the corresponding author.

## Supplementary Materials

The following supporting information can be downloaded at: https://www.mdpi.com/article/10.3390/nano12132127/s1, Figure S1: Schematic illustration of fabrication process of the BLA CNTs/SEBS pressure sensor on PI substrate; Figure S2: SEM images of BLA CNTs layer as a variation of incident BL energy density; Figure S3: Optical images of SEBS thickness variation on BLA CNTs; Figure S4: Fabrication process flow of stretchable BLA CNTs/SEBS sensor.
