# Supplementary material for: Highly Sensitive, Stretchable Pressure Sensor Using Blue Laser Annealed CNTs
Source: Nanomaterials (Basel). 2022 Jun 21;12(13):2127. doi: 10.3390/nano12132127 (PMC9268723; doi:10.3390/nano12132127)
Supplement: Supplementary file 1 [file nanomaterials-12-02127-s001.zip › nanomaterials-1760941-supplementary.pdf]

nanomaterials

Supplementary Material

Highly Sensitive, Stretchable Pressure Sensor Using Blue Laser Annealed CNTs

Chanju Park, Munsu Choi, Suhui Lee, Hyunho Kim, Taeheon Lee, Mohammad Masum Billah, Byunglib Jung and Jin Jang *

Advanced Display Research Center (ADRC), Department of Information Display, Kyung Hee University, 26 Kyungheedae-ro, Dongdaemun-gu, Seoul 02447, Korea; cjpark@tft.khu.ac.kr (C.P.); mschoi@tft.khu.ac.kr (M.C.); shlee5@tft.khu.ac.kr (S.L.); hhkim@tft.khu.ac.kr (H.K.); thlee@tft.khu.ac.kr (T.L.); masum@tft.khu.ac.kr (M.M.B.); bljung@tft.khu.ac.kr (B.J.)

* Correspondence: jjang@khu.ac.kr

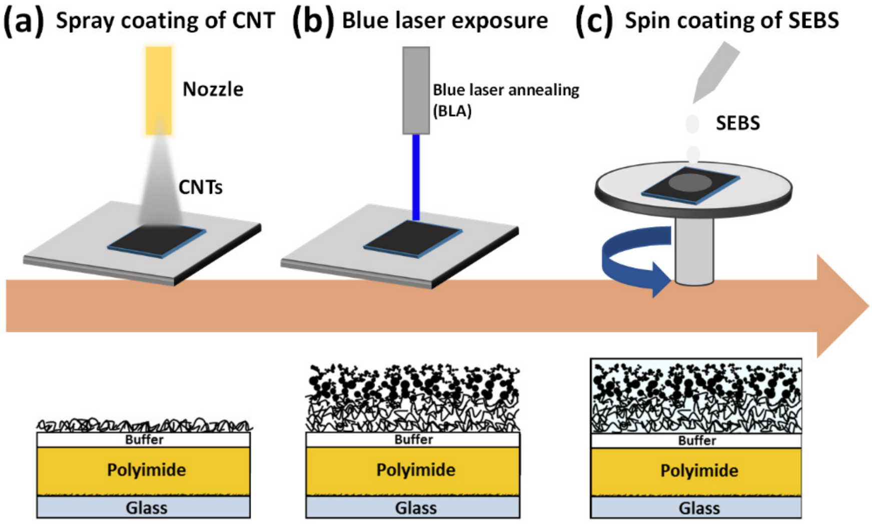

Figure S1. Schematic illustration of fabrication process of the BLA CNTs/SEBS pressure sensor on PI substrate. (**a**) spray-coating of CNTs, (**b**) blue laser annealing, and (**c**) SEBS coating on BLA CNTs layer.

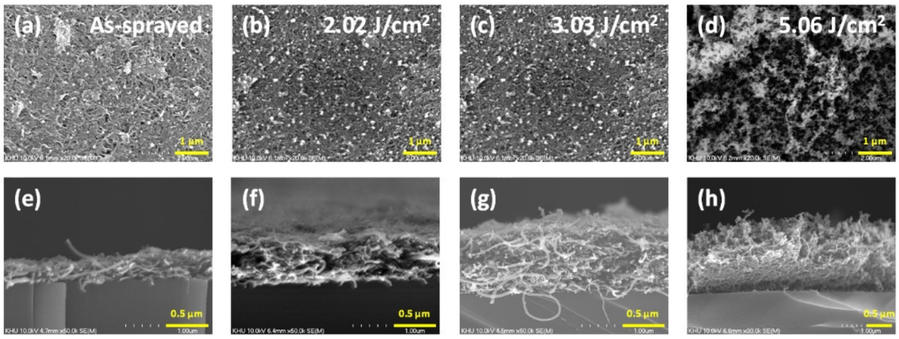

Figure S2. SEM images of BLA CNTs layer as a variation of incident BL energy density. (**a**)–(**d**) Top and (**e**)–(**h**) cross-sectional SEM images of the BLA CNTs as a variation of BLA energy density: (**a**), (**e**) 0, (**b**), (**f**) 2.02, (**c**), (**g**) 3.03, and (**d**), (**h**) 5.06 J cm^{-2}.

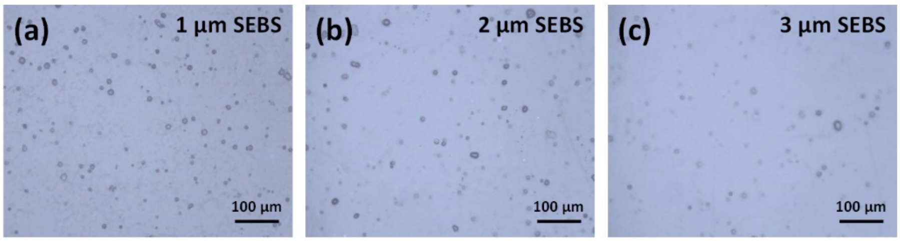

Figure S3. Optical images of SEBS thickness variation on BLA CNTs. (**a**) 1 μm, (**b**) 2 μm, and (**c**) 3 μm.

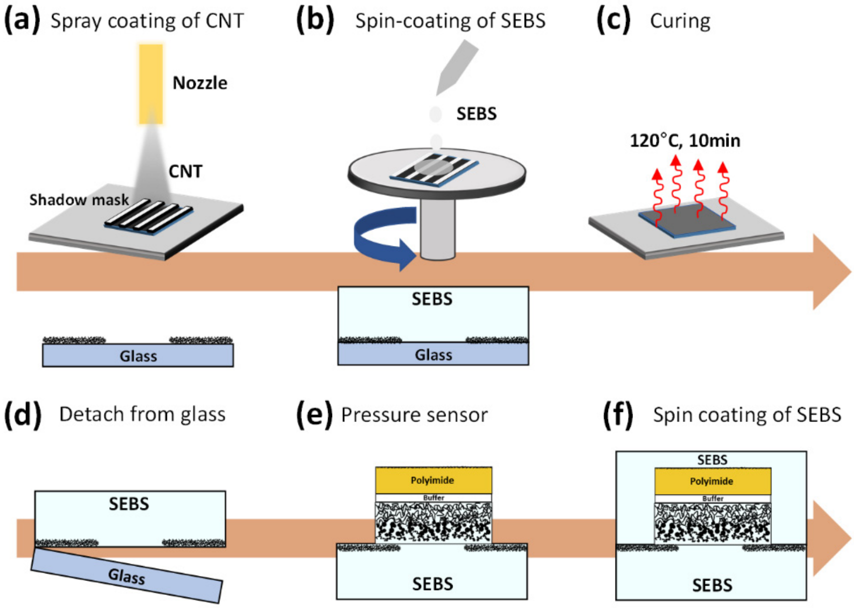

Figure S4. Fabrication process flow of the (**a**)–(**d**) stretchable electrodes and (**e**, **f**) stretchable pressure sensor. (**a**)–(**e**) Fabrication of stretchable electrodes on SEBS substrate and the detachment of stretchable electrodes from the carrier glass and (**e**) transfer of pressure sensor on the stretchable CNT electrodes on SEBS substrate. (**f**) Pressure sensor covered with SEBS for passivation.
